# Model organisms in POLG-related disorders: insights from yeast to multicellular systems

**DOI:** 10.1038/s41419-025-08366-6

**Published:** 2025-12-26

**Authors:** Raquel Brañas Casas, Giovanni Risato, Alessandro Zuppardo, Carlo Viscomi, Francesco Argenton, Mara Doimo, Nicola Facchinello, Natascia Tiso

**Affiliations:** 1 Department of Biology, University of Padova, Padova, Italy; 2 Department of Women’s and Children’s Health, University of Padova, Padova, Italy; 3 Pediatric Research Institute (IRP) - Fondazione Città della Speranza, Padova, Italy; 4 Department of Biomedical Sciences, University of Padova, Padova, Italy; 5 Veneto Institute of Molecular Medicine (VIMM), Padova, Italy; 6 Department of Pharmacy and Biotechnology, University of Bologna, Bologna, Italy; 7 Neuroscience Institute, Italian Research Council (CNR), Padova, Italy

**Keywords:** Zebrafish, Experimental models of disease

## Abstract

Mitochondrial genetic diseases are complex disorders that impair cellular energy production, leading to diverse clinical manifestations across multiple organ systems. These diseases arise from mutations in either mitochondrial DNA or nuclear DNA. Among nuclear DNA-related cases, mutations in *POLG* and *POLG2*, which encode subunits of mitochondrial DNA polymerase γ, are particularly significant, causing conditions such as Alpers–Huttenlocher syndrome and progressive external ophthalmoplegia. Model organisms have been instrumental in elucidating POLG-related disease mechanisms and advancing therapeutic strategies. *Saccharomyces cerevisiae* (budding yeast) provided insights into fundamental mitochondrial functions, while *Caenorhabditis elegans* (roundworm) helped explore POLG’s roles in multicellular organisms. *Drosophila melanogaster* (fruit fly) has been pivotal in studying neurological aspects, and *Mus musculus* (mouse) models contributed to understanding systemic effects in mammals. Recently, *Danio rerio* (zebrafish) has emerged as a promising vertebrate model for drug screening, due to its optical transparency and genetic tractability. Each model system offers unique advantages, collectively bridging the gap between basic research and clinical applications. This review will examine in vivo models used in POLG disorder research, highlighting their contributions to understanding disease mechanisms and therapeutic advancements.

## Facts

- The review highlights the lack of approved therapies specifically targeting POLG-related mitochondrial disorders, underscoring an urgent need for interventions.
- Various model organisms, from yeast to roundworm, fruit fly, zebrafish, and mice, have been utilised to characterise disease phenotypes and mechanisms of POLG/POLG2 disorders, each offering unique advantages.
- The availability of these diverse models enables a multi-species approach for screening drugs and potential therapies against POLG-related diseases, including repurposing FDA-approved compounds.

## Introduction

### Mitochondria and mitochondrial DNA

**Fig. 1:**
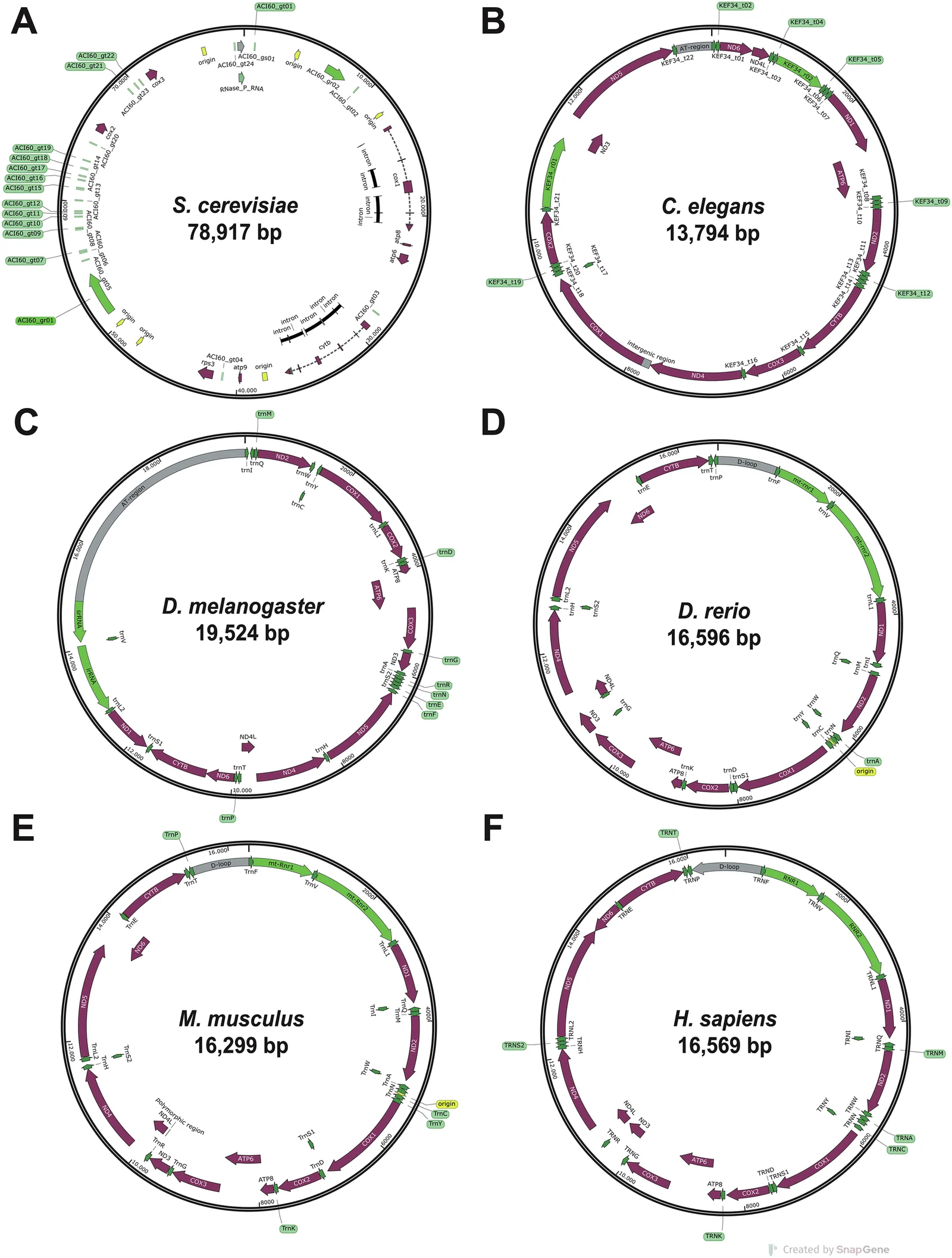
Comparative mitochondrial genomes of representative eukaryotes. Comparison of the mitochondrial genomes of *S.* *cerevisiae* (**A**), *C. elegans* (**B**), *D. melanogaster* (**C**), *D. rerio* (**D**), *M. musculus* (**E**), and *H. sapiens* (**F**). Annotated DNA sequences were obtained from the GenBank^®^ database (https://www.ncbi.nlm.nih.gov/genbank/), and the genetics maps were generated using SnapGene software.

**Fig. 2:**
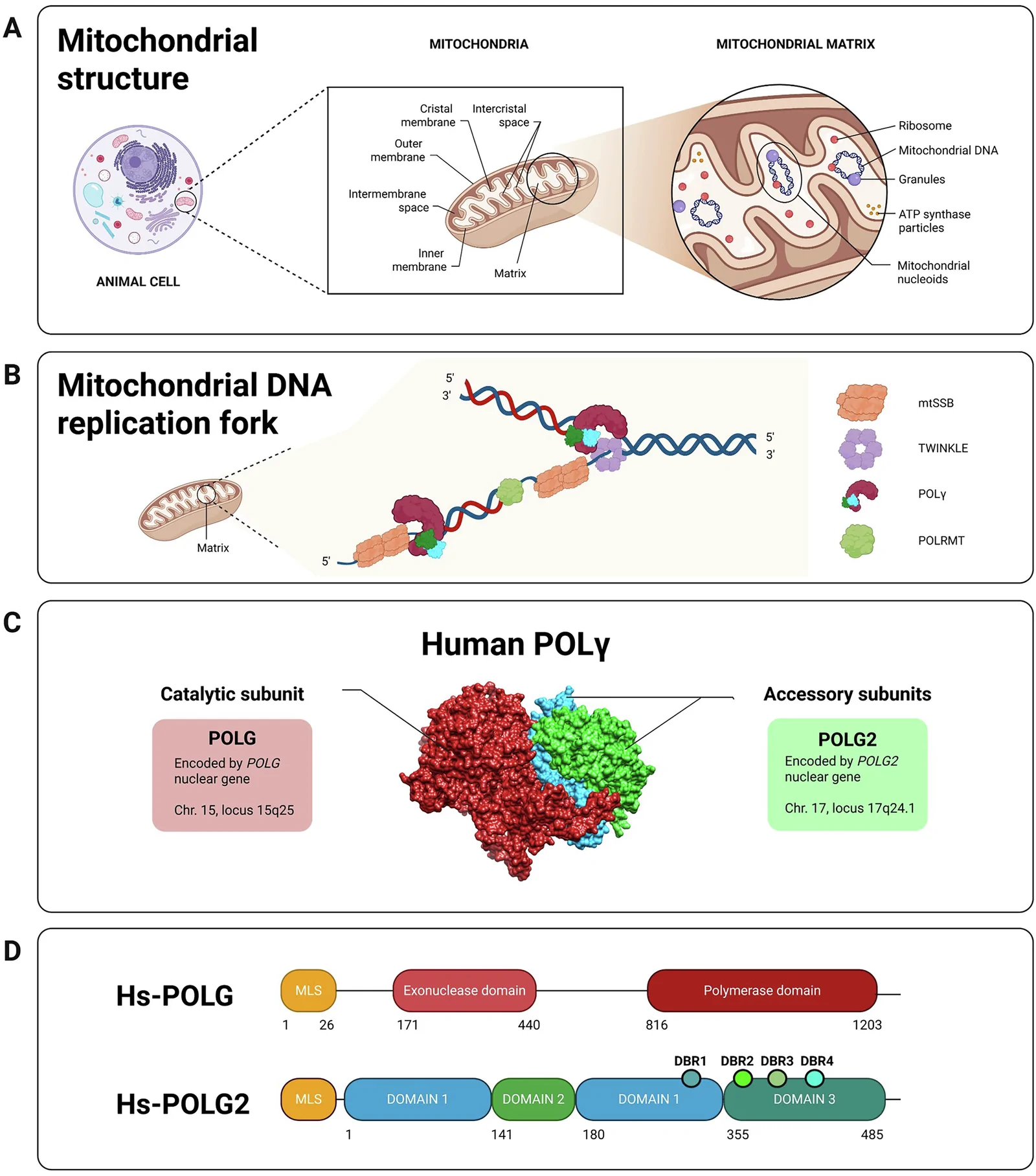
Mitochondria: structure, replication and human POLγ subunits. **A** Representation of the traditional “cristae junction model” of a mitochondrion from an animal cell. The mtDNA is located in the mitochondrial matrix. Adapted from Logan et al. (2006), BioRender (2020), and Huang, E. (2023) [88, 89]. **B** The mitochondrial DNA replication fork. Schematic representation of mtDNA packaged into nucleoids by the mitochondrial replisome. POLRMT, a mitochondrial RNA polymerase, acts as a primase. Modified from Filograna et al. (2021) [90]. **C** Human POLγ 3D model. Structure obtained from PDB (3IKM) and modified with Chimera. **D** Diagrams of the human catalytic subunit of POLγ (top), adapted from Rahn et al. (2015) [50], and the human accessory subunit of POLγ (bottom), adapted from Wojtaszek et al. [91]. Created with BioRender.

Mitochondria, discovered nearly two centuries ago, are essential organelles in eukaryotic cells. They play crucial roles in cellular metabolism, primarily producing ATP through oxidative phosphorylation (OXPHOS) [1]. Human mitochondrial DNA (mtDNA), a circular molecule of 16,569 base pairs, encodes 37 genes, including 13 proteins essential for the OXPHOS system, 22 tRNAs, and 2 rRNAs [2] (Fig. 1). Unlike nuclear DNA, mtDNA replicates continuously and independently of cell division [3]. The minimal mtDNA replisome consists of the DNA polymerase-gamma (POLγ) holoenzyme (composed of POLG and POLG2 subunits), which is essential for replication and repair, the TWINKLE helicase for DNA unwinding, and mtSSB for stabilising single-stranded DNA [4–6] (Fig. 2).

### POLG-related disorders and mitochondrial DNA maintenance

POLG-related disorders are the most common and well-studied among mtDNA maintenance defects (MDMDs) [7]. These disorders result from mutations in either *POLG* (chromosome 15q25) or *POLG2* (chromosome 17q24.1) genes, which encode the catalytic (p140 or POLγA or POLG) and accessory subunits (p55 or POLγB or POLG2) of POLγ, respectively [8, 9].

Mutations in the *POLG* gene can disrupt POLγ function, leading to mtDNA instability through accumulation of multiple deletions, point mutations, or copy number depletion. More than 300 *POLG* pathogenic mutations are listed in the Human DNA Polymerase Gamma Mutation Database [10]. These genetic lesions impair mitochondrial function, resulting in a spectrum of diseases whose clinical manifestations depend on mutation type, the extent of mtDNA instability, and the tissues affected [7, 11–13]. Childhood myocerebrohepatopathy spectrum (MCHS) is an early-onset disorder characterised by encephalopathy, developmental delay, myopathy, hypotonia, and liver failure [14]. Alpers–Huttenlocher syndrome (AHS), typically presenting between 2 and 4 years of age, involves intractable epilepsy, psychomotor regression, liver disease, ataxia, and neuropathy [15, 16]. Myoclonic epilepsy myopathy sensory ataxia (MEMSA) usually has a juvenile onset and is associated with cerebellar ataxia, epilepsy, encephalopathy, and myopathy [15]. Progressive external ophthalmoplegia (PEO), which occurs in both dominant (adPEO) and recessive (arPEO) forms, is a late-onset disease that affects extraocular muscles, causing ptosis and ophthalmoparesis [7].

POLG-related disorders can also result from mutations in POLG2, the accessory subunit of POLγ. The ClinVar NCBI database reports more than a dozen *POLG2* mutations. Unlike *POLG* mutations, *POLG2* variants do not appear to compromise the fidelity of mtDNA synthesis [9, 17]. Dominant *POLG2* mutations typically cause adPEO, often accompanied by additional symptoms such as skeletal muscle weakness, ataxia, depression, and progressive neurosensory issues [18]. The first reported adPEO case caused by a *POLG2* mutation was a missense variant in the dimerisation domain, c.1352G>A (p.Gly451Glu) [10]. Such mutations can lead to mtDNA replication fork stalling, causing deletions and depletion over time [10, 17]. Recessive *POLG2* mutations have also been identified. A homozygous c.544C>T p.(Arg182Trp) mutation was found in a case of early-onset hepatic failure [19], affecting POLG2 dimerisation [20]. Another homozygous variant, c.1297G>T (p.Asp433Tyr), was reported in an adult with optic atrophy, movement disorders, premature ovarian failure, and mtDNA depletion [21].

### Model organisms for POLG-related disorders

Various model organisms—including yeast, worms, fruit flies, zebrafish, and mice—have been developed to study POLG-related disorders. The structure and function of POLγ differ across eukaryotes: it is monomeric in yeast and nematodes, heterodimeric in insects, and heterotrimeric in vertebrates, underscoring its evolutionary significance in mitochondrial function [22] (Table 1).

**Table 1:** General overview of POLγ in different model organisms, evaluating their strengths and limitations.

Species	Catalytic subunit			Accessory subunit			Strength & weakness	Phenotypic comparison
	Gene & form	Protein length (AA)	Human % Identity/Similarity	Gene & form	Protein length (AA)	Human % Identity/Similarity		
*S. cerevisiae*	*MIP1* monomeric	1259	45 / 61	-			+Low-cost model, fast-growing, and ideal for large-scale drug screening. - Lacks accessory subunit (POLG2). Limited evolutionary conservation and no multicellular context.	Exhibits mitochondrial dysfunction, defects in the proofreading activity of replicative polymerases and increased mtDNA mutability.
*C. elegans*	*polg-1* monomeric	1072	37/51	–			+Low-cost model, easy to maintain, transparent body. Enables live imaging. Useful for high-throughput genetic screens. - Lacks accessory subunit and shows lower homology to human POLG.	Displays mitochondrial dysfunction, developmental delay, reduced fertility, locomotor defects, shortened lifespan and features of premature aging.
*D. melanogaster*	*PolG1* monomeric	1145	44/58	*PolG2* monomeric	361	25/42	+Invertebrate model with partially conserved POLG2 subunit. + Powerful genetic tools and short lifecycle for rapid studies. - Only moderate sequence homology and possible functional redundancy. - Monomeric POLG2 subunit.	Loss of POLG in *Drosophila melanogaster* causes a mitochondrial mutator phenotype, leading to neurodegeneration, reduced lifespan, locomotor defects, and sterility, partially overlapping with human POLG-related mitochondrial syndromes.
*D. rerio*	*polg* monomeric	1206	70/79	*polg2* dimeric	442	47/67	+ Vertebrate system with heterotrimeric POLG complex conserved as in humans. + Transparent embryos suitable for both in vivo functional studies (organ-level phenotypes) and drug screening. - More complex to manipulate at adult stages.	Zebrafish *polg/polg2* mutants share a core set of phenotypes, including severe mitochondrial DNA depletion, slowed growth and developmental delay, reduced mitochondrial respiration, disorganised skeletal muscle with impaired motility, cardiac defects, altered liver and brain development, activation of hypoxia-related signalling, and premature death.
*M. musculus*	*Polg* monomeric	1217	87/90	*Polg2* dimeric	459	79/88	+ High sequence homology to human POLG and POLG2. + Availability of knock-in/ knock-out models. + Phenotypes observed at multiple system levels - Expensive, time-consuming, and unsuitable for high-throughput screening. - Embryonic lethality. - Ethical limitations.	Mouse *Polg* models recapitulate multiple clinical features of human POLG disorders: develop mitochondrial DNA instability leading to premature aging, muscle and bone degeneration, impaired locomotion, cardio-myopathy, neurodegeneration, and activation of mitochondrial stress responses.
*H. sapiens*	*POLG* monomeric	1239	100	*POLG2* dimeric	485	100	+ Patient-derived iPSCs enable modelling of mutation-specific effects and drug response. + Suitable for CRISPR-based correction and pharmacological testing. - Limited availability of patient tissue and high variability among genetic backgrounds.	iPSC-based systems reveal mitochondrial dysfunction, impaired oxidative phosphorylation, and mtDNA instability, offering a precise platform for therapeutic development.

Protein sequences were retrieved from Ensembl database and the *Saccharomyces* Genome Database (SGC), then aligned using the BLAST bioinformatics tool. Species lacking the accessory subunit are indicated with “–”.

Mutants in POLG or POLG2 have been generated using diverse strategies, including gene knockdown (KD), knock-out (KO), transgenic overexpression (Tg), site-directed mutagenesis, chemically or ENU-induced mutagenesis, and knock-in (KI) of human variants. A detailed analysis of these mutant models is provided in the following chapters and summarised in Table 2.

**Table 2:** Summary table of all generated POLG and POLG2 mutants in different model organisms.

Mutated gene	Protein	Technique	Model organism	Reference
*Polg*^*D181A*^ Asp181Ala	DNA polymerase gamma catalytic subunit	Site-directed mutagenesis	Mouse	Zhang et al. (2000)
*PolG2*	DNA polymerase gamma accessory subunit	Chemical mutagenesis	Fly	Iyengar et al. (2002)
*Polg*^*D257A*^ Asp257Ala	DNA polymerase gamma catalytic subunit	Knock-in model mutator mouse	Mouse	Trifunovic et al. (2004)
*Polg*	DNA polymerase gamma catalytic subunit	Knock-out model	Mouse	Hance et al. (2005)
*Polg*^*D181A*^ Asp181Ala, CaMKIIα promoter	DNA polymerase gamma catalytic subunit	Transgenic mice	Mouse	Kasahara et al. (2006)
*mip1Δ*	Mip1 (DNA polymerase gamma)	Site-directed mutagenesis	Yeast	Baruffini et al. (2006)
*Polg* (α-MyHC/Y955C)	DNA polymerase gamma catalytic subunit	α-MyHC^30^ transgenic mice	Mouse	Lewis et al. (2007)
*polg-1*	DNA polymerase gamma	International *C. elegans* gene knock-out consortium	Worm	Bratic et al. (2009)
*Polg2*	DNA polymerase gamma accessory subunit	Conditional knock-out	Mouse	Humble et al. (2013)
*polg*	DNA polymerase gamma catalytic subunit	Knock-out model	Zebrafish	Rahn et al. (2015)
*polg-1(srh1)*	DNA polymerase gamma	CRISPR-Cas9 knock-in mutator worm	Worm	Haroon et al. (2018)
*Polg2*^*Y265X*^ Tyr265Stop	DNA polymerase gamma accessory subunit	ENU mutagenesis	Mouse	Gorvin et al. (2019)
*PolG1*	DNA polymerase gamma catalytic subunit	CRISPR-Cas9 knock-out	Fly	Marygold et al. (2020)
*polg*	DNA polymerase gamma catalytic subunit	Morpholino, knock-out model & ENU mutagenesis	Zebrafish	Facchinello et al. (2021)
*Polg*^*A449T*^ Ala449Thr	DNA polymerase gamma accessory subunit	Knock-in model	Mouse	Silva-Pinheiro et al. (2021)
*PolG2*	DNA polymerase gamma accessory subunit	RNAi-mediated knock-down	Fly	Rodrigues et al. (2022)
*Polg*^*W726S*^ Trp726Ser	DNA polymerase gamma catalytic subunit	Knock-in model	Mouse	Kang et al. (2024)
*polg2*	DNA polymerase gamma accessory subunit	Knock-out model	Zebrafish	Brañas Casas et al. (2024)
*iSkM-Polg*^*mut*^	DNA polymerase gamma catalytic subunit	Conditional knock-out	Mouse	Bond et al. (2025)
*Polg*^*R292C*^ Arg292Cys	DNA polymerase gamma catalytic subunit	Knock-in model	Mouse	VanPortfliet et al. (2025)
*Polg*^*G826S*^ Gly826Ser	DNA polymerase gamma catalytic subunit	Knock-in model	Mouse	Corrà et al. (2025)
*Polg*^*Y933C*^ Tyr933Cys	DNA polymerase gamma catalytic subunit	Knock-in model	Mouse	Corrà et al. (2025)

#### Saccharomyces cerevisiae

*Saccharomyces cerevisiae* (budding yeast) is a valuable model for studying POLG-related disorders due to its functional conservation with human mitochondrial genes. Of note, its anaerobic metabolism allows survival with an inactivated OXPHOS system [23, 24]. Yeast mtDNA is predominantly linear, ranging from 68 to 86 kb, with 10–200 copies per nuclear genome [25].

The yeast mitochondrial genome is replicated by Mip1, a monomeric mitochondrial DNA polymerase that shares 43% sequence homology with human POLG (Table 1). Mip1 retains key domains including mitochondrial targeting signal (MTS), exonuclease, linker, and polymerase regions [24–27]. Despite lacking an accessory subunit, yeast compensates through shortened protein subdomain sequences [28].

*S. cerevisiae* has been crucial in elucidating the pathogenicity of POLG mutations, their underlying molecular defects, and the influence of genetic background [27]. For example, it helped characterise the Tyr955Cys and Gly268Ala variants as dominant mutations affecting mtDNA replication and mutability [28]. This model has proven excellent for validating human POLG mutations’ pathogenicity by introducing them into Mip1 and analysing their effects on yeast growth and viability [29].

#### Caenorhabditis elegans

*Caenorhabditis elegans*, a free-living nematode (roundworm) first identified in England in 1900, has become an excellent model for studying mitochondrial disorders due to its short life cycle, ease of manipulation, and conserved mitochondrial functions [30].

Unlike *S. cerevisiae*, *C. elegans* mtDNA structure closely resembles its human counterpart, comprising multiple 13.7 kb circular molecules with overlapping gene content. The mitochondrial genome has been fully sequenced [30]. These worms exhibit heteroplasmy, and mtDNA copy numbers fluctuate throughout their life cycle [31]. Mitochondrial genome maintenance is essential for worm development, which arrests when mtDNA replication is blocked [30, 31].

*C. elegans* has been crucial in understanding mitochondrial dynamics, inheritance, and mitophagy [32]. It compensates for the lack of nucleotide excision and mismatch repair machinery with an efficient base excision repair mechanism, correcting mistakes caused by ROS accumulation [32, 33].

*polg-1*-deficient worms, carrying mutations in their monomeric mitochondrial DNA polymerase, develop normally until adulthood but exhibit reduced mtDNA content, shortened lifespan, and sterility [34]. This was the first model organism shown to complete embryonic and larval development in the absence of active POLγ. In *C. elegans*, mtDNA copy number remains stable until the L3 larval stage, after which it increases exponentially during the transition to adulthood. In *polg-1* mutants, however, mtDNA levels are reduced, resulting in impaired gonadal function [34]. These findings suggest that while mtDNA copy number maintenance is critical for normal development, compensatory mechanisms enable *polg-1* mutant worms to progress through embryogenesis and larval stages even in the absence of the mitochondrial replicase [34].

Addo et al. established an innovative platform using *C. elegans* to identify new genes responsible for mtDNA maintenance and discover novel orthologues of human genes associated with MDMDs [30]. This was a breakthrough in linking target genes to mitochondrial disease.

A *polg-1* (ok1548/+) model, carrying a 2149 bp deletion in the polymerase domain, was analysed by Pitayu et al. (2016) [35]. Heterozygous mutants exhibited reduced egg deposition (brood size) but retained normal behaviour. These findings further support that *C. elegans* can survive with few mitochondrial genomes, although higher copy numbers are required for energy-demanding processes, such as reproduction [31, 34, 35].

More recently, Haroon et al. (2018) used CRISPR-Cas9 to introduce the Asp207Ala substitution in worms (*polg-1(srh1)*), mimicking the *PolgA*^*D257A*^ mutator mouse. This mutant displayed both mtDNA depletion and elevated mutagenesis due to defective POLγ activity [36].

#### Drosophila melanogaster

The fruit fly *Drosophila melanogaster* provides an advanced platform to study polymerases in a complex multicellular organism. Its short life cycle, small size, rapid reproduction, and easy maintenance make it an ideal model. Although mtDNA gene content is conserved between flies and humans, the genetic sequences diverge, with fruit fly mtDNA displaying a higher A-T content [37].

Unlike the heterotrimeric POLγ complex in vertebrates, the mitochondrial replicase in *Drosophila* is a heterodimer [22]. The first purification of the fruit fly PolG complex in 1986 revealed this heterodimeric structure [38]. PolG has since been well characterised in *D. melanogaster*, and its role in mtDNA replication remains a major research focus [22, 37].

Multiple *Drosophila* mutants have been generated to investigate POLG function. Mutations in the catalytic subunit PolG1 (*tam* mutants) cause premature death, altered replisome assembly, reduced mtDNA content, and visual system defects [39]. PolG1-deficient flies are also weaker and show delayed development [34].

Notably, *D. melanogaster* was the first animal model used to study the POLG accessory subunit; PolG2-null mutants exhibit severe phenotypes including pupal lethality, mtDNA loss, and impaired cell proliferation [39].

Overall, the *Drosophila* model has been instrumental in elucidating mechanisms of mtDNA maintenance, uncovering a role for ROS in regulating mtDNA copy number, and advancing our understanding of mitochondrial diseases. Nevertheless, mechanistic differences between flies and humans must be taken into account when extrapolating findings to POLG-related disorders.

#### Danio rerio

*Danio rerio* (zebrafish), a tropical freshwater fish native to South Asia, has emerged as an excellent model organism for studying vertebrate biology, development, genetics, and human disease since its introduction to biological research in the 1960s [40].

As a vertebrate, the zebrafish is more closely related to humans than invertebrate models such as *C. elegans* and *D. melanogaster*, while remaining easier to manipulate genetically and embryologically than mammalian models [41]. It offers several advantages, such as small body size, cost-effective maintenance, and the presence of most organs relevant to human diseases [42]. Females can spawn weekly, releasing hundreds of transparent eggs, which enable easy monitoring throughout developmental stages [41, 43, 44]. This natural translucency, combined with fluorescence-based transgenic tools, permits in vivo visualisation and analysis of mitochondrial distribution and dynamics across different tissues [45], making zebrafish a valuable in vivo model for studying mitochondrial pathophysiology.

The zebrafish genome is fully sequenced and well-annotated (GRCz11, 2017), sharing around 70% of protein-coding genes with humans [46].

Various technologies have been developed for genetic manipulation in zebrafish, including antisense Morpholino oligomers (MOs), zinc finger nucleases (ZFNs), transcription activator-like effector nucleases (TALENs), CRISPR-Cas9 system, and N-Ethyl-N-nitrosourea (ENU) mutagenesis [47, 48].

Zebrafish mtDNA shares ~70% sequence identity with human mtDNA, with both genomes encoding 37 genes [49]. Zebrafish possess homologues of the human *POLG1* and *POLG2* genes and a heterotrimeric POLγ complex. Unlike mice, *polg* mutants in zebrafish recapitulate many phenotypes observed in human POLG patients [50]. The first *polg* mutants in zebrafish were full knock-outs (*polg*^*muz119*^, *polg*^*muz120*^), resulting in significant mtDNA depletion, delayed development, impaired energetics, and embryonic lethality [50]. Subsequently, an adult-viable ENU-induced *polg* nonsense point mutation (*polg*^*sa9574*^) was characterised, with homozygous mutants developing mtDNA depletion and other POLG-like phenotypes, including cardiac, skeletal muscle, hepatic, and gonadal defects [51]. A larval-lethal CRISPR-Cas9-generated microdeletion (*polg*^*ia302*^) was also briefly studied [51].

The first zebrafish *polg2* KO line (*polg2*^*ia304*^) was generated using CRISPR-Cas9, carrying a 10-nucleotide deletion in exon 4 of the *polg2* gene. This mutant line exhibited phenotypes similar to Polg mutants, including severe mtDNA depletion, altered mitochondrial dynamics, reduced growth, impaired locomotor activity, and premature death [52].

These zebrafish models have effectively recapitulated many clinical characteristics observed in human patients with POLG-related disorders. The studies underscore the critical role of both catalytic (Polg) and accessory (Polg2) components of POLγ in zebrafish mtDNA replication and organism survival, as homozygous mutations in *polg* and *polg2* severely impair larval development and lifespan.

#### Mus musculus

*Mus musculus*, a rodent native to the Indian subcontinent, shares ~99% of its genes with humans, making it a highly relevant model for pre-clinical studies and therapeutic development [53, 54]. The mitochondrial genome of most inbred laboratory mice is derived from *M. m. domesticus* [55]. Mouse models have been instrumental in elucidating the mechanisms of mtDNA maintenance and their links to disease [56].

Mouse POLγ closely resembles the human holoenzyme, with up to 90% similarity in both catalytic and accessory subunits (Table 1), making it suitable for studying human MDMDs and *POLG/POLG2* pathogenic variants. The first *Polg* null mutant, generated by Hance et al. (2005), revealed that homozygous mutants die between E7.5 and E8.5, demonstrating POLG’s essential role in mammalian embryonic development. These mutants were smaller and exhibited delayed development compared to heterozygous and wild-type siblings [57, 58]. Lewis et al. (2007) developed a transgenic mouse line overexpressing the Tyr955Cys POLG mutation in heart tissue. This resulted in decreased lifespan, mtDNA depletion, and various cardiac abnormalities [58, 59]. Zhang et al. (2000) generated a transgenic mouse overexpressing the p.Asp181Ala (*Polg*^*D181A*^) variant in the heart [60], leading to mtDNA mutations and deletions observable through cardiomyopathy [58]. Kasahara et al. (2006) created a neuron-specific Asp181Ala POLG transgenic mouse, resulting in mood disorder-like symptoms and altered monoaminergic functions [56, 61]. Trifunovic et al. (2004) and Kujoth et al. (2005) developed the “mutator” mouse model carrying the *Polg*^*D257A*^ allele [62, 63], exhibiting premature aging phenotypes and reduced lifespan [58]. Silva-Pinheiro et al. (2021) presented a knock-in mouse model reproducing the common human recessive mutation Ala467Thr, which impairs DNA binding and reduces POLγ efficiency [64]. Kang et al. (2024) generated a mouse model carrying the homologous human mitochondrial recessive ataxia syndrome (MIRAS) allele, displaying normal lifespan but decreased POLG protein levels, compromised mtDNA replication, and altered sensitivity to tick-borne encephalitis virus infection [65]. More recently, Bond et al. (2025) described a tissue-specific *Polg* mutant displaying mitochondrial dysfunction and muscle degeneration, with robust activation of the mitochondrial integrated stress response (mtISR) pathway [66]. Corrà et al. (2025) generated two mouse *knock-in* models mimicking the human Gly848Ser and Tyr955Cys pathogenic variants [67]. Their work highlighted both similarities and differences between human and murine polymerase, opening new possibilities for therapeutic interventions. In addition, they produced compound heterozygous mice with allelic combinations associated with AHS in humans, identifying one mutant (*Polg*^*A449T/G826S*^) with marked mtDNA depletion in high-energy-demanding tissues. VanPortfliet et al. (2025) reported a model harbouring a variant corresponding to the human Arg309Cys mutation, which displayed elevated IFN-I responses and hyper-inflammation following *Pseudomonas aeruginosa* challenge [66–68].

Regarding POLG2, Humble et al. (2013) characterised the first *Polg2* KO mice, revealing that homozygous mutants died during embryogenesis, while heterozygous siblings were unaffected [69]. Gorvin et al. (2019) induced a nonsense mutation (Tyr265Stop) in the *Polg2* gene using ENU mutagenesis, showing that a single copy of the mutant allele was sufficient to alter mtDNA levels in heterozygous mice [70].

In conclusion, mouse models have proven invaluable for studying POLG-related disorders, disease progression, and therapeutic development, as the molecular pathways and pathogenetic mechanisms are conserved between mice and humans. However, it is important to note that functional knockouts of *POLG* or *POLG2* in vertebrates cause nearly complete loss of mtDNA and lethality before adulthood [50, 57, 69].

### Human cellular models

Induced pluripotent stem cells (iPSCs) have recently been used to study human *POLG* mutations. Typically derived from patients’ fibroblasts, iPSCs can carry either homozygous or heterozygous *POLG* variants, although they often display only partial phenotypes [71]. Liang and colleagues also developed a protocol to differentiate neural stem cells (NSCs) from patient-derived iPSCs. The NSC model recapitulated the molecular and biochemical hallmarks of POLG dysfunction observed in patient brain tissues. Notably, compound heterozygous *POLG* condition produced more severe phenotypes, including impaired energy production, mtDNA depletion, and accumulation of ROS, reflecting key aspects of POLG-related disorders [72].

Another application of iPSCs in POLG-related research has been the generation of cortical organoids that recapitulate human phenotypes [73]. This system represents a major advance for therapeutic screening, particularly for disorders caused by impaired neuronal mtDNA maintenance. Treatment of POLG organoids with metformin rescued observed phenotypes, improving morphology, restoring neuronal markers, and increasing mitochondrial mass, thereby validating POLG organoids as a valuable preclinical platform [73].

While iPSC-based approaches closely mimic physiological conditions, they remain in vitro systems and cannot fully capture the complexity of human disease [74]. Patient-derived iPSCs allow correlations between specific mutations and their functional consequences, enabling targeted drug screening. However, limited tissue availability and variability in patient genetic backgrounds remain major limitations. As an alternative, CRISPR-Cas-based iPSC models have been employed to generate POLG neuronal models of diseases with isogenic controls [74].

Despite various strategies to enhance iPSC applications over the years, only a few studies have directly addressed *POLG2* mutations, leaving the accessory subunit poorly understood. Within this research field, the work of Do and colleagues has helped clarify the role of POLG2 in the POLγ complex. Specifically, by performing *POLG2* KO in cybrids (cytoplasmic hybrids with mitochondria from one cell and nuclear background from another), they found that POLG2 is required to maintain mtDNA and stabilise the catalytic subunit POLG [75].

### From yeast to multicellular models: a translational approach in POLG drug discovery

Drug repurposing—the strategy of identifying new therapeutic uses for already approved medications—has emerged as an efficient alternative to traditional drug discovery. By exploiting the known pharmacological and safety profiles of existing drugs, this approach can significantly reduce the time, cost, and complexity typically associated with de novo drug development [76–78]. As a result, libraries of FDA-approved drugs are increasingly being explored for new therapeutic opportunities [79, 80].

In the context of POLG-related mitochondrial diseases, a multi-model screening approach has been used to identify promising drug candidates. Pitayu and colleagues employed *Saccharomyces cerevi*siae to evaluate nearly 1500 FDA-approved compounds for their ability to rescue respiratory defects caused by Mip1 mutations [35]. Molecules showing beneficial effects were designated as MIP1 rescuing substances (MRSs). This screen led to the identification of clofilium tosylate (CLO) as a potential therapeutic agent, with validated activity also in other POLG models, including *Caenorhabditis elegans* and patient-derived fibroblasts [35]. However, further testing in whole vertebrate animals was necessary before advancing toward human application.

To bridge the gap between unicellular/invertebrate models and vertebrate systems, zebrafish have emerged as a valuable vertebrate model for drug screening. Their sensitivity to physiologically relevant drug doses and the ease of compound administration via the aqueous environment make them particularly suitable for high-throughput assays [79, 81]. Small molecules can diffuse directly into embryos, crossing the blood-brain barrier if non-polar, thereby enabling efficient phenotypic screening [78]. Exploiting these advantages, CLO was tested in zebrafish models carrying mutations in *polg* and *polg2*, where it successfully rescued several POLG-associated phenotypes [51, 52]. Collectively, these findings supported the utility of a yeast-zebrafish pipeline for the preclinical assessment of candidate drugs targeting POLG disorders caused by either gene, prior to validation in mammalian models, including advanced systems such as CRISPR-engineered large animals.

Beyond model organisms, an encouraging breakthrough was recently reported directly in patient-derived cellular systems. Valenzuela and colleagues identified PZL-A—a small molecule known to activate mtDNA synthesis—as a potential therapeutic agent. Their study showed that PZL-A can both stimulate wild-type POLγ in vitro and restore mtDNA levels in cells from patients with POLG-related disorders. Specifically, fibroblasts carrying well-characterised *POLG* mutations were first treated with ethidium bromide to exacerbate mtDNA depletion and then incubated with PZL-A. Treatment with PZL-A improved POLγ-DNA-binding stability and, consequently, restored POLγ complex function across multiple pathogenic mutations [82].

## Discussion

The scarcity of experimental systems addressing POLG and POLG2 dysfunction has long hindered the modelling and pharmacological treatment of POLG-related phenotypes. This review aimed to bridge that gap by examining various model organisms and therapeutic strategies designed to counteract POLG/POLG2 defects.

The yeast system, represented by *mip1*-deficient *Saccharomyces cerevisiae* strains, was among the first models shown to recapitulate key POLG-associated phenotypes observed in human patients. The unicellular nature of yeast also makes it particularly well-suited for large-scale drug screening. Loss of POLG in *Saccharomyces cerevisiae* results in a mitochondrial DNA mutator phenotype, characterised by defective proofreading and accumulation of mutations [25, 83]. Although Mip1 lacks an accessory subunit, it contains intrinsic domains that functionally compensate for the role of POLG2 in humans. However, low sequence identity with human POLG and the absence of a multicellular context limit its translational relevance.

*Caenorhabditis elegans*, despite lacking an accessory subunit, has proven effective for drug screening in a simplified multicellular context, benefiting from low-cost maintenance and genetic similarity to humans. The *polg-1* gene encodes the catalytic subunit, and mutant or knockdown strains display mitochondrial dysfunction, developmental delays, and motility defects. Still, the overall phenotype is relatively mild, and the organism’s evolutionary distance from humans constrains direct pathological comparisons [30, 84].

*Drosophila melanogaster* offers an intriguing avenue for exploring POLG2 function, serving as an invertebrate model in which POLG2 is partially conserved. The availability of strong genetic tools and the presence of both catalytic and accessory subunits make it a valuable platform for studying protein interactions and domain function. Mutants in *PolG1* and *PolG2* show neurodegenerative phenotypes, locomotor impairment, and shortened lifespan, features partially overlapping with human disease. However, only moderate sequence conservation and possible functional redundancy may limit its utility for mechanistic studies at the protein level [34, 85].

*Danio rerio* (zebrafish) has emerged as a powerful vertebrate model for POLG-related disorders, combining optical transparency, external development, and genetic tractability. Zebrafish retain the heterotrimeric organisation of the POLG complex found in humans and display major organ phenotypes relevant to disease. Mutants for *polg* and *polg2* recapitulate key pathological features, from mitochondrial DNA depletion to multi-organ dysfunction. Loss of *polg2* is typically more severe and often lethal, whereas hypomorphic *polg* alleles enable long-term disease studies [50–52]. These models are particularly valuable for translational research, including in vivo drug screening. Importantly, because mutations in both genes impair brain development and function, zebrafish provide a unique opportunity to explore the neurological manifestations of POLG-related diseases—such as encephalopathy, epilepsy, and ataxia—which remain incompletely understood and represent a key direction for future investigation.

*Mus musculus*, although physiologically relevant, has limited application in high-throughput drug screening, as existing POLG mutants often present either embryonic lethality or only mild phenotypes. Instead, it serves as a valuable model for mechanistic studies of disease progression. Mutant mice exhibit progressive mtDNA depletion, neurodegeneration, and premature aging, closely mirroring POLG-related mitochondrial diseases. Nevertheless, their limited utility in large-scale screening, combined with high maintenance costs and ethical constraints, render them suboptimal for early-stage drug discovery [66, 67, 86].

Finally, human iPSC-derived models offer a patient-specific in vitro system that directly captures the genetic background of POLG-related disorders. These models allow the study of mutation-specific effects on mtDNA maintenance, respiratory function, and response to therapeutic compounds. Moreover, they provide an ideal platform for genome editing and drug repurposing efforts, although variability between lines and limited scalability remain important challenges.

The use of *mip1*-deficient *S. cerevisiae*, *C. elegans polg-1* strains, *D. rerio polg/polg2* mutants, and patient-derived cell lines has successfully led to the identification of POLG-rescuing compounds, many of which are FDA-approved drugs repurposed for mitochondrial disorders. These advances underscore the complementary nature of diverse model systems: while no single model fully recapitulates the human condition, together they form a robust experimental pipeline for unravelling disease mechanisms and accelerating therapeutic development for POLG-related mitochondrial pathologies.

## Conclusions

POLG-related disorders are among the more than 7000 rare diseases that currently lack approved treatments. Therapies for mitochondrial diseases remain largely limited to symptom management, underscoring the urgent need for interventions that restore mtDNA stability. This review highlights the critical role of model organisms in drug screening for POLG disorders.

Drug repurposing presents a time-efficient and cost-effective alternative to the development of novel therapeutics [87]. A high-throughput screen in Mip1-deficient yeast identified candidate molecules—predominantly FDA-approved—capable of rescuing POLG-related phenotypes across multiple model systems [35, 51, 52]. These studies demonstrated that drugs identified through POLG-targeted screens—initially in yeast and subsequently validated in *C. elegans* and *D. rerio*—have the potential to mitigate POLG/POLG2-dependent phenotypes in humans, pending further validation in mammalian systems. Collectively, these findings support the use of a multi-species approach as a robust platform for drug discovery in POLG-related and other mitochondrial DNA depletion disorders.
