# Correction: Model organisms in POLG-related disorders: insights from yeast to multicellular systems

**DOI:** 10.1038/s41419-026-09022-3

**Published:** 2026-08-31

**Authors:** Raquel Brañas Casas, Giovanni Risato, Alessandro Zuppardo, Carlo Viscomi, Francesco Argenton, Mara Doimo, Nicola Facchinello, Natascia Tiso

**Affiliations:** 1 Department of Biology, University of Padova, Padova, Italy; 2 Department of Women’s and Children’s Health, University of Padova, Padova, Italy; 3 Pediatric Research Institute (IRP)—Fondazione Città della Speranza, Padova, Italy; 4 Department of Biomedical Sciences, University of Padova, Padova, Italy; 5 Veneto Institute of Molecular Medicine (VIMM), Padova, Italy; 6 Department of Pharmacy and Biotechnology, University of Bologna, Bologna, Italy; 7 Neuroscience Institute, Italian Research Council (CNR), Padova, Italy

**Keywords:** Zebrafish, Experimental models of disease

Correction to: *Cell Death & Disease* https://doi.org/10.1038/s41419-025-08366-6, published online 26 December 2025

In this article, the author list Brañas Casas R, Risato G, Zuppardo A, Viscomi C, Argenton F, Doimo M, Facchinello N, Tiso N. has been published erroneously as Casas RB, Risato G, Zuppardo A, Viscomi C, Argenton F, Doimo M, Facchinello N, Tiso N.

And the first author’s name should be interpreted as follows:

- Given name: Raquel
- Family name: Brañas Casas (double surname)

Therefore, the correct full name is Raquel Brañas Casas, where Brañas Casas constitutes the full family name.

The original article has been corrected.

